# Genetic Polymorphisms and Genetic Risk Scores Contribute to the Risk of Coronary Artery Disease (CAD) in a North Indian Population

**DOI:** 10.3390/ijms25158552

**Published:** 2024-08-05

**Authors:** Sarabjit Mastana, Kushni Charisma Halai, Liz Akam, David John Hunter, Puneetpal Singh

**Affiliations:** 1 School of Sport, Exercise and Health Sciences, Loughborough University, Loughborough LE11 3TU, UKe.c.akam@lboro.ac.uk (L.A.); d.j.hunter@lboro.ac.uk (D.J.H.); 2 Department of Human Genetics, Punjabi University, Patiala 147002, India; puneetpalsingh@pbi.ac.in

**Keywords:** CAD, North India, polygenic risk score, GST*M1, GST*T1, *ACE*, *AGT* M235T, *AGT* T174M, *AGTR1* A1166C, *APOE*, *APOA5*, haplotypes

## Abstract

Coronary artery disease (CAD) is the leading cause of death in India. Many genetic polymorphisms play a role in regulating oxidative stress, blood pressure and lipid metabolism, contributing to the pathophysiology of CAD. This study examined the association between ten polymorphisms and CAD in the Jat Sikh population from Northern India, also considering polygenic risk scores. This study included 177 CAD cases and 175 healthy controls. The genetic information of *GSTM1* (rs366631), *GSTT1* (rs17856199), *ACE* (rs4646994), *AGT* M235T (rs699), *AGT* T174M (rs4762), *AGTR1* A1166C (rs5186), *APOA5* (rs3135506), *APOC3* (rs5128), *APOE* (rs7412) and *APOE* (rs429358) and clinical information was collated. Statistical analyses were performed using SPSS version 27.0 and SNPstats. Significant independent associations were found for GST*M1, GST*T1, *ACE*, *AGT* M235T, *AGT* T174M, *AGTR1* A1166C and *APOA5* polymorphisms and CAD risk (all *p* < 0.05). The *AGT* CT haplotype was significantly associated with a higher CAD risk, even after controlling for covariates (adjusted OR = 3.93, 95% CI [2.39–6.48], *p* < 0.0001). The *APOA5/C3* CC haplotype was also significantly associated with CAD (adjusted OR = 1.86, 95% CI [1.14–3.03], *p* < 0.05). A higher polygenic risk score was associated with increased CAD risk (adjusted OR = 1.98, 95% CI [1.68–2.34], *p* < 0.001). Seven polymorphisms were independently associated with an increase in the risk of CAD in this North Indian population. A considerable risk association of *AGT*, *APOA5/C3* haplotypes and higher genetic risk scores is documented, which may have implications for clinical and public health applications.

## 1. Introduction

Coronary artery disease (CAD), also termed coronary heart disease, is the leading cause of mortality and the loss of Disability Adjusted Life Years (DALYs) worldwide [1]. CAD is a non-communicable disease that is multifactorial and polygenic [2]. Research has shown that certain populations, notably South Asians (people of the Indian sub-continent), have an increased susceptibility to CAD [2]. The highest rates of the premature onset of CAD are amongst native and overseas Indians, occurring 10 years earlier than in other ethnicities [3]. In 2016 in India, 17.8% of the total deaths were attributable to CAD, with an increase in the prevalence of CAD from 25.7 million in 1990 to 54.5 million in 2016 [4].

Chronic injury to the coronary arteries leads to the development of atherosclerotic plaques due to factors such as lipid deposits, sheer stress, genetic alterations, and oxidative injury. This results in an inflammatory response and thickening of arterial walls, eventually restricting blood flow to the heart. CAD develops and progresses due to the interplay of modifiable (e.g., smoking, hypertension, diabetes, dyslipidaemia, obesity) and non-modifiable factors (age, sex, ethnicity, family history) as well as an individual’s genetic makeup [5]. Genome-wide (GWASs) and candidate-gene association studies have identified a large range of single-nucleotide polymorphisms (SNPs) and genetic loci that are associated with an increased risk of developing CAD [6,7,8]. However, the findings are inconsistent across populations and ethnicities.

This study focuses on the analysis of selected CAD candidate genes or GWAS-identified loci to assess their contribution to the risk of the development of CAD in a North Indian population. Ten polymorphisms were chosen for this study and details are provided in Table 1. These polymorphisms belong to three main categories of direct and indirect contributors to the CAD: (a) Glutathione S-transferases (GSTs) are a family of isoenzymes which regulate cellular detoxification against exogenous toxins, oxidative stress and DNA damage [9]; (b) the Renin–angiotensin–aldosterone system (RAAS) regulates arterial blood pressure by increasing water, salt reabsorption, and vascular tone and dysfunction of the system can result in hypertension and CAD risk [10], and (c) Apolipoproteins which regulates lipoprotein metabolism, plasma triglyceride (TG), LDL levels and total cholesterol levels [11].

The primary aim of this study is to evaluate whether the ten genetic polymorphisms are associated with CAD risk in the Jat Sikh population from Northern India. The secondary aim is to investigate whether the accumulation of risk alleles, termed the polygenic risk score (PRS), increases the risk of CAD.

## 2. Results

Clinical, demographic, and biochemical parameters of controls and CAD cases are presented in Table 2. Age (*p* < 0.001) and SBP (*p* = 0.002) were significantly higher among CAD cases than controls and HDL, DBP, cholesterol, LDL and total lipids were significantly higher among controls (*p* < 0.001). There was no significant difference between both groups for sex, BMI, smoking status or TG. There were significant differences in the prevalence of higher diabetes, higher hypertension and lower HDL levels in cases (all *p* values < 0.05).

### 2.1. Genotype and Allele Distribution and Association Analysis

The genotype and allele frequencies and the HWE significance are presented in Table 3. All the polymorphisms, where HWE could be assessed, did not deviate from HWE.

For all the polymorphisms studied, the odds for the association of the risk of CAD in the multiplicative model (allelic model) were greater than 1, even after adjusting for demographic, SBP, DBP, and lipid parameters (except *APOC3* rs5128) (Table 4), but not all of them were statistically significant. A significant association with CAD risk was observed in seven polymorphisms, and results remained significant after adjustments. All the polymorphisms involved in the RAAS (*ACE* rs4646994, *AGT* rs699, *AGT* rs4762 and *AGTR1* rs5186) were significantly associated with an approximately 2-fold increase in the risk of CAD in all association models, before and after adjustments. For *ACE* rs4646994 and *AGTR1* rs5186, the adjusted ORs were greater than the crude ORs under all association models. The *AGTR1* rs5186 C/C genotype carried the greatest risk of CAD. The null genotypes of GST*M1 and GST*T1 were associated with a 2.78-fold and a 2.12-fold increased risk of developing CAD. *APOA5* rs3135506 was significantly associated with the susceptibility to CAD under the multiplicative, dominant and co-dominant (C/C vs. G/G) models at crude levels, but only remained significant for the allelic model after controlling for demographic and lipids. No significant association was found in any of the association models for *APOE* rs7412, *APOE* rs429358 and *APOC3* rs5128.

### 2.2. Linkage Disequilibrium and Haplotype Analysis

The strongest linkage disequilibrium result was observed in the *AGT* rs699-rs4762 haplotype presented with the strongest disequilibrium result, with it being coinherited 89% of the time, and it was the only result that was statistically significant (D′ = 0.891, r = 0.870, r^2^ = 0.756, *p* < 0.001). The *APOE* rs7421-rs429358 haplotype presented with a very weak linkage disequilibrium, and the results suggest that the polymorphisms are coinherited 41% of the time, and the results were not statistically significant (D′ = 0.410, r = −0.035, r^2^ = 0.001, *p* = 0.354). The weakest linkage disequilibrium was observed in *APOA5/C3* rs3135506-rs5128, with the polymorphisms being coinherited 3% of the time (D′ = 0.030, r = 0.024, r^2^ = 0.001, *p* = 0.530).

The distribution of haplotype frequencies among controls and CAD cases and the odds of CAD susceptibility are presented in Table 5. For *AGT* SNPs, the haplotype CT was found to carry a 340% increased risk of developing CAD in the study sample, independently of age, sex and BMI. A smaller adjusted risk for CAD was found in the CC haplotype of *APOA5/C3* SNPs (OR = 1.57, 95% CI [1.01–2.45]). No significant associations were found for other haplotypes.

### 2.3. Polygenic Risk Score

**Figure 1:**
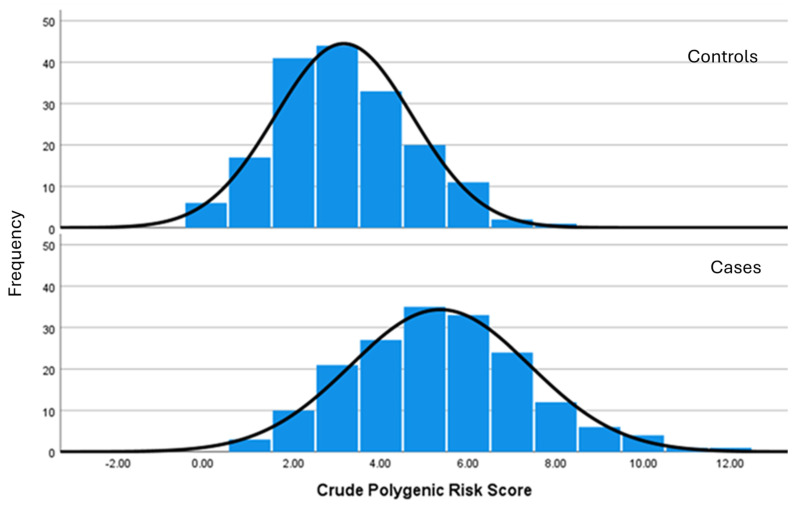
Polygenic risk score distribution among controls and CAD cases.

The highest crude PRS was 12 in the CAD cases group. No participants were homozygous for the risk alleles for all the polymorphisms analysed. Figure 1 shows the distribution of PRSs amongst controls and CAD cases. The results of the Mann–Whitney U test showed that the crude PRS of the 10 polymorphisms was significantly different in the CAD cases compared to the controls (5.00 (4.00–7.00) vs. 3.00 (2.00–3.00), *p* < 0.001). After adjusting for demographic and lipid parameters, significant associations were found between the crude PRS (OR = 1.98, 95% CI [1.68–2.34], *p* < 0.001) and the risk of developing CAD in this population.

**Figure 2:**
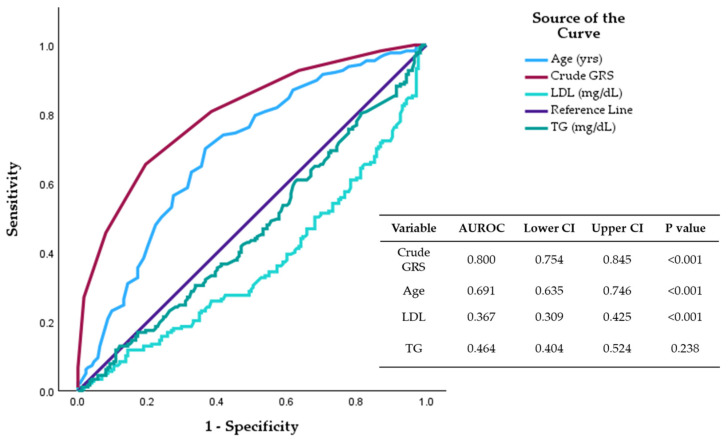
Receiver operating characteristic (ROC) for crude GRS, age, LDL and TG.

The crude GRS/PRS area under the curve (AUC) was a significantly better predictor (0.80 CI 0.75–0.84, *p* < 0.001) of the CAD risk compared to other clinical and demographic variables in this population (Figure 2).

## 3. Discussion

This comprehensive study of an endogamous North Indian population analysed ten polymorphisms that play major roles in the pathophysiology of CAD. The results document a significant association of seven genetic polymorphisms in the development of CAD. The analysis of clinical and biochemical parameters showed that DBP, cholesterol, LDL and total lipids were lower in the CAD group compared to the control group. This is unexpected as high DBP and lipid profiles are risk factors for CAD. This discrepancy could be confounded by hypertensive and lipid-lowering medication, which were not accounted for when data were collected.

### 3.1. GSTM1 and GSTT1

Lipid peroxidation and the accumulation of free radicals due to oxidative stress are known to be involved in the pathogenesis of cardiovascular diseases including CAD. GST protects cells against these by catalysing the conjugation of glutathione (GSH) to a wide variety of both endogenous and exogenous electrophilic molecules. The null genotype produces no mu-1 or theta-1 enzyme decreasing conjugative capacity and hence increasing the risk of CAD [9]. In this present study, *GSTM1* rs366631 polymorphism was significantly associated with CAD and remained significant after adjusting for classical risk factors (null genotype OR = 2.78, 95% CI [1.79–4.33], *p* < 0.0001). The null genotype was almost two times more frequent in CAD cases (52%) compared to controls (28%). These results provide more insight into the role of this polymorphism among the population of India and strengthens previously reported results on the potential role of this polymorphism in CAD [13,34].

Only a few studies have assessed the risk association of *GSTT1* and CAD in India. This study found that the rs17856199 polymorphism was significantly associated with CAD (null genotype OR = 2.12, 95% CI [1.28–3.52], *p* = 0.005). A meta-analysis documented a significant association between GST*T1 and CAD risk in South Asians (OR = 1.81, 95% CI [1.04–3.14], *p* = 0.03) but not in East Asians (OR = 1.05, 95% CI [0.85–1.29], *p* = 0.65) and Europeans (OR = 1.09, 95% CI [0.91–1.31], *p* = 0.35) [35] suggesting the diversification of roles of GST polymorphisms among different populations. Despite this possible divergence in the role of GST polymorphisms in CAD within different ethnic groups there is renewed interest in oxidative stress and inflammatory status in the context of CAD. This is evidenced by a developing interest in medications for CAD which may modulate the inflammatory and oxidative stress balance such as colchicine [36,37].

### 3.2. The RAAS Genetic Polymorphisms

The RAAS genetic polymorphisms (*ACE* rs4646994, *AGT M235T* rs699, *AGT T174M* rs4762 and *AGTR1 A1166C* rs5186) were associated with CAD in the present study, which suggests that these genes play a significant role in the pathogenesis of CAD. *ACE* rs4646994 was associated with an increased risk of developing CAD under all association models and was larger after adjustments. The D/D genotype carried the greatest risk compared to the I/I genotype (adjusted OR = 4.57, 95% CI [2.22–9.24], *p* = 0.0004). These results support the findings of a recent study in North India that showed a significant association of the *ACE* D/D genotype with CAD risk (OR = 1.81, 95% CI [1.22–2.66], *p* = 0.003) [38]; however, Agrawal et al. [39] reported no association with CAD in a different regional population.

The *AGT* rs699 C allele was significantly associated with CAD (adjusted OR = 3.66, 95% CI [2.11–5.35], *p* < 0.0001). Additionally, after adjusting for covariates, there was an approximate 5-fold increased risk in individuals with the C/C genotype compared to the T/T genotype. Consistently with these findings, the rs699 polymorphism was significantly associated with an increased risk in an Iranian population [19]. Due to the limited number of studies investigating the independent association of *AGT* rs699 and CAD susceptibility in India, further research needs to be conducted to confirm the results of this study.

There is limited evidence of the association between AGT rs4762 and CAD within the Indian population. One study found no significant association between this polymorphism and CAD [40]. This contradicts the results of the present study, which observed a significant association with CAD under all models, suggesting that carriers of the T allele have an increased risk of developing CAD in the Punjab population of India. The discrepancies may be due to sample size differences or sampling from different regions of India.

The TT haplotype of *AGT* rs699 and *AGT* rs4762 had a greater frequency in the CAD group (30.4%) than in the controls (9.1%). An association analysis showed that having the risk haplotype TT significantly increases the risk of CAD by more than 4-fold and remains significant after adjusting for covariates.

The greatest risk association between the polymorphisms and CAD was with *AGTR1* rs5186 and CAD after adjusting for covariates (OR = 10.15, 95% CI [3.14–32.84], *p* < 0.0001). Under all association models, the odds increased after adjustments, suggesting a greater independent association of rs5186 with CAD. However, Mishra et al. [41] found no significant association between the C allele and C/C genotype with the risk of CAD in the North Indian population. Thus, the role of *AGTR1* rs5186 and CAD susceptibility in India requires further investigation. Nevertheless, the C allele was associated with an increased risk of CAD in East Asia [25] and the Chinese population [26,27]. The association ORs reported by Zhang, Zhou and Zhang (2012) [25] were smaller than those seen in the present study. This may suggest that the role of *AGTR1* rs5186 in the pathogenesis of CAD could be greater in Indian populations than in Chinese populations.

### 3.3. Apolipoprotein Genetic Polymorphisms

There was no significant association observed in both polymorphisms of *APOE* (rs7412 and rs429358) with an increased risk of CAD. This could be due to no individual harbouring the T/T genotype of rs7412, so the frequency of the T allele is small. Further research with a larger sample size could confirm this. Although the majority of studies on *APOE* gene report an association of the ε2, and ε4 alleles with CAD risk, only a few studies present the distribution and association of the C and T alleles of *APOE* rs7412 and *APOE* rs429358. Takeuchi et al. [42] found a positive association between rs7412 and CAD susceptibility (OR = 1.69, 95% CI [1.46–1.95], *p* = 6.1 × 10^−13^) but no significant association for rs429358 (OR = 0.95, 95% CI [0.87–1.04], *p* = 0.240).

A haplotype analysis of *APOE* rs7412 and *APOE* rs429358 found no statistical significance in any combination. The CT haplotype represents the ε3 allele of *APOE* and presented with the highest frequency in both groups (CAD: 0.822; controls: 0.792), as expected [43]. The CC haplotype represents the ε4 allele and the TT haplotype represents the ε2. Studies have shown the ε4 increased the risk of CAD in the Kashmiri population of India (OR = 2.04, 95% CI [1.46–2.85], *p* < 0.001) [44] and in other populations [33,45]. This contradicts the present study’s findings and further research on the association of the *APOE* gene with CAD risk in this North Indian population is warranted.

ApoA5 has been suggested to regulate TG metabolism, and if it is deficient, then TG levels increase [46]. An analysis adjusted for age, sex, BMI and lipid parameters showed a significant 59% increased risk of developing CAD with *APOA5* rs3135506 C allele. However, Bhanushali and Das [47] found no significant association of *APOA5* rs3135506 with CAD (OR = 0.56, 95% CI [0.220–1.442], *p* = 0.267). This could be attributable to a lower statistical power due to the relatively small sample size. Another study also reported no significant risk association with CAD under the multiplicative model (OR = 0.59, 95% CI [0.1–1.9], *p* = 0.384) [30]. These results could be explained by the low frequency of the risk allele in the study population.

No significant association was observed between *APOC3* rs5128 and CAD in this study. Similar findings have been reported in other studies evaluating the association among Indian populations [10,47,48]. In contrast, a meta-analysis of 31 studies found a significant association of rs5128 with CAD risk under the multiplicative model in the overall population (OR = 1.14, 95% CI [1.05–1.24], *p* = 0.003) [49]. This contradicts the findings in Indian populations, suggesting that there is a difference in the role of this polymorphism in the pathogenesis of CAD between ethnic groups.

*APOA5* and *APOC3* are located in the gene cluster APOA1/C3/A4/A5, so an haplotype analysis was conducted. Functional apoAV and apoCIII result in opposite roles in TG metabolism [46]. The results found that carrying the CC haplotype of *APOA5* rs3135506 and *APOC3* rs5128 increases CAD susceptibility by approximately 60%, even after adjusting for covariates. This suggests that functional apoCIII and apoAV deficiency increases CAD risk, which supports the emerging evidence of lipid metabolism and CAD susceptibility [46].

### 3.4. Polygenic Risk Score

Although not all the studied polymorphisms were significantly associated independently with CAD, the results suggest that the accumulation of the risk alleles increases the risk. There was a 98% increased risk of CAD with the crude GRS/PRS, demonstrating the polygenic nature of the disease. Genetic risk scores have been created from many GWASs to provide insight for it to be an independent risk predictor of CAD [50,51]. Although the ROC curve analysis revealed that the GRS was a stronger predictor of CAD status than other variables in this population, further comprehensive genomic analyses are required. Caution is warranted in the interpretations as known clinical indicators (LDL/TG) were below the reference line possibly due to lipid-lowering medications in patients. A study in a Portuguese population analysed the risk associated with CAD of 31 loci using genetic risk scores. It found that the highest quartile of weighted PRS was associated with a significant increase in the risk of developing CAD (OR = 2.588, 95% CI [2.090–3.204], *p* < 0.0001) [21]. Shahid et al. [52] analysed the genetic risk score of 21 polymorphisms and also found a significant risk associated with possessing more than 19 risk alleles compared to possessing fewer than 13 (OR = 2.96, 95% CI [1.71–5.13], *p* < 0.001). Further studies need to be performed to determine the validity and reliability of PRS as a risk predictor of CAD.

### 3.5. Limitations and Further Research

There are some limitations to this study that should be considered. Firstly, the statistical power is limited due to the relatively small sample size. Secondly, this population is endogamous, resulting in homozygosity. This would result in the over- or underrepresentation of genotypes, which again limits the statistical power. However, the genotypic distribution of all the polymorphisms was in HWE, so it can be assumed that the results of this study are representative of the general population. Thirdly, due to the case–control design of the present study, a causal relationship could not be established between the polymorphisms and CAD. Finally, information on the medication being taken by the participants was not collected, which is a possible confounding factor. As it has been shown that the blood lipid profile influences *APOE* polymorphisms and CAD susceptibility [53], this could explain the lack of an association with CAD risk observed in *APOE* rs7412 and rs429358 and in *APOC3* rs5128. Further research should be conducted on the polymorphisms analysed in this study in other Indian populations to confirm their role in CAD susceptibility. With endogamy being common in India, studies in sub-populations may provide a better insight into the differences in CAD prevalence and mortality rates in the different states. These studies should aim to recruit a larger sample size for sufficient statistical power. They should also attempt to control or adjust for medication that influences biochemical parameters, which could provide greater statistical power. Furthermore, since significant associations were found in the polymorphisms that play a role in the RAAS, studies could be carried out to assess how to target this system through lifestyle factors or medication to reduce the increasing burden of CAD in India.

## 4. Materials and Methods

This case–control study was approved by the Loughborough University Ethical Approvals (Human Participants) sub-committee (LEON ID 1233). All 352 participants from the Jat Sikh population from Punjab (Patiala and Kapurthala districts), separated by 3 generations, provided written consent for their participation in the study and publication of the collated results. The Jat Sikh community is a dominant agriculturist group in the Punjab state of North India and comprises approximately 21% of the population. Their lifestyle is agrarian and mostly non-vegetarian and professing to the Sikh religion. This study adhered to the Declaration of Helsinki principles. Healthy controls were selected based on having no previous history or current symptoms of CAD or metabolic disorders. The control group comprised 175 individuals (104 males and 71 females) with an average age of 54.6 ± 12.6 years. The case (patients) group included 177 individuals (112 males and 65 females) with an average age of 62.9 ± 11.6 years. CAD cases were classified based on at least 50% or more stenosis in one or more coronary arteries [54]. The clinical diagnosis was performed by experienced cardiologists using coronary angiography. The sample size calculation using software package Quanto version 1.2 [55] estimated that a sample of 170 patients and 170 controls should be sufficient to detect an odds ratio of 2.0 or above at 80% power using allele frequency information from previous studies.

Blood samples were analysed in the laboratory without the knowledge of disease status. Demographic data were collected on age and sex. Clinical and biochemical data were collected on body mass index (BMI), smoking status, systolic blood pressure (SBP), diastolic blood pressure (DBP), cholesterol, TG, LDL, HDL, and total lipids. Genotyping and biochemical parameter measures were carried out as per standard methods [54]. GST*M1, GST*T1, rs4646994 loci were analysed using specific PCR primers followed by gel electrophoresis. Other loci (rs699, rs4762, rs5186, rs7412, rs429358, rs3135506 and rs5128) were analysed using the TaqMan-based QPCR technique. The laboratory analysis was repeated for 10% of the samples for genotyping to ensure repeatability and validity of obtained results.

Statistical Package for the Social Sciences (SPSS) IBM software (version 27.0) was used to calculate descriptive statistics of clinical, demographic and biochemical parameters between the groups. Age was the only continuous parameter that was normally distributed, so it is expressed as mean ± standard deviation (SD), and a comparison between groups was analysed using Student’s *t*-test. Continuous parameters that were not normally distributed were expressed as the median (interquartile range), and comparisons were tested using a Mann–Whitney U test. Categorical parameters were expressed as a number (percentage) and Chi-squared test or Fisher’s exact test was used to analyse differences between groups.

Microsoft Excel was used to collate all genetic and clinical data. Since *GSTM1* and *GSTT1* only had two outcomes (null and wild) in the dataset, deviation from the Hardy–Weinberg equilibrium (HWE) could not be determined. HWE, crude ORs, adjusted ORs and their 95% CIs were calculated for the multiplicative (allelic), dominant and recessive association models of the other polymorphisms using SNPStats (https://www.snpstats.net/start.htm, last accessed on 30 June 2024) [56]. A linkage disequilibrium (*D*′ and *r* statistics) and haplotype analysis between rs699-rs4762, rs7412-rs429358 and rs3135506-rs5128 were conducted on SNPStats. Crude odds ratios (ORs) and the 95% confidence interval (CI) for the null alleles of *GSTM1* and *GSTT1* with CAD status were calculated using Excel (version 2406). Bonferroni-corrected *p* value (0.05/10 loci = 0.005) was used to correct for multiple comparisons.

Microsoft Excel was also used to calculate crude risk scores. For each polymorphism, a risk score of 0, 1 or 2 was given depending on the number of risk alleles present. The crude PRS was the sum of the number of risk alleles an individual possesses. The Mann–Whitney U test was used to compare the PRS between groups since the results were not normally distributed. The results were presented as the median (interquartile range). The *p* value < 0.05 was considered statistically significant.

## 5. Conclusions

In conclusion, this study showed that the *GSTM1*, *GSTT1*, *ACE*, *AGT* M235T, *AGT* T174M and *AGTR1* A1166C polymorphisms increase the risk of CAD in the Jat Sikh population, in Northern India. Additionally, the expression of more than one risk allele for multiple polymorphisms is a risk factor for CAD. To further solidify the significance of these findings, case–control studies with larger sample sizes of sub-populations in India should be conducted. Furthermore, PRS and its ability to predict CAD requires validation for it to be used clinically.

## Figures and Tables

**Table 1:** Details of polymorphisms analysed in this study that may influence the risk of CAD.

Gene	SNP/Variant	Variant Type	Cytoband	Alleles	Description of Variant	CAD Risk	Supporting Studies	Contradicting Studies
*Glutathione S-transferase mu 1 (GSTM1)*	rs366631	Deletion	1p13.3	null/wild	Homozygous deletion (null) leads to the absence of the enzyme. Non-deletion allele carriers produce a function enzyme.	↑	Su et al., 2020 [9] Song et al., 2021 [12]	
*Glutathione S-transferase theta 1 (GSTT1)*	rs17856199	Deletion	22q11.23	null/wild	Homozygous deletion (null) leads to the absence of the enzyme. Non-deletion allele carriers produce a function enzyme.	↓	Bhatti et al., 2018 [13]	Song et al., 2017 [14]
*Angiotensinogen-converting enzyme (ACE)*	rs4646994	Insertion/deletion	17q23.3	I/D	Located in intron 16 of *ACE*, it is an insertion (I) or deletion (D) of a 287bp *Alu* repeat.	↑	Moradzadegan et al., 2015 [15]	Kondo et al., 2015 [16]
							Temel et al., 2018 [17]	Bonfim-Silva et al., 2016 [18]
*Angiotensinogen (AGT)*	rs699	Exonic	1q42.2	M/T (T/C)	Transition from T-to-C at nucleotide 704 of exon 2 results in substituting threonine for methionine at amino acid 235. It is also known as M235T.	↑	Khatami et al., 2017 [19] Zhao et al., 2020 [20]	Bonfim-Silva et al., 2016 [18] Pereira et al., 2018 [21]
	rs4762	Exonic	1q42.2	C/T	Methionine substitutes threonine at amino acid 174, due to the transition of C-to-T at nucleotide 521 on exon 2. It is also known as T174M.	↑	Li et al., 2012 [22] Khatami et al., 2017 [19]	Wang, 2013 [23] Jia et al., 2012 [24]
*Angiotensinogen receptor type 1 (AGTR1)*	rs5186	Exonic	3q24	A/C	Also known as A1166C, it is a transversion at nucleotide 1166 from A-to-C in the 3′ untranslated region of the *AGTR1*.	↑	Zhang et al., 2012 [25] Tian et al., 2015 [26] Duan and Wang, 2016 [27]	Pereira et al., 2018 [21]
*Apolipoprotein A-V (APOA5)*	rs3135506	Exonic	11q23.3	G/C	Also known as S19W, is the substitution of tryptophan from serine, due to a G-to-C transversion.	↑	Morjane et al., 2020 [28]	Zhou et al., 2013 [29]
								Kashyap et al., 2018 [30]
*Apolipoprotein C-III (APOC3)*	rs5128	3′UTR	11q23.3	C/G	Transversion at nucleotide 3238 from C-to-G, located within the 3′ untranslated region of *APOC3*.	↑	Li et al., 2016 [31]	Rai et al., 2016 [11]
*Apolipoprotein E (APOE)*	rs7412	Exonic	19q13.32	C/T	These two variants combined create the three alleles ε2, ε3 and ε4. If the rs429358 allele is T and the same chromosome has the rs7412 T allele, ε2 is created. If the same chromosome harbours both C alleles, then ε4 is formed. ε3 is created by the rs7412 C allele and the rs429358 T allele.	ε2: ↓ ε4: ↑	Nordlie et al. 2005 [32] Xu et al., 2016 [33]	
	rs429358	Exonic	19q13.32	T/C				

SNP: single-nucleotide polymorphism; ↑: susceptible association; ↓: protective association; 3′UTR: 3′ untranslated region.

**Table 2:** Clinical, demographic and biochemical characteristics of patients and controls from Jat Sikh population, North India.

Parameter	Controls (*n* = 175)	Cases (*n* = 177)	*p* Values
Age (years)	54.63 ± 12.63	62.94 ± 11.54	<0.001 *
Sex			
Male	104 (59%)	112 (63%)	0.512
Female	71 (41%)	65 (37%)	
BMI	26.21 (23.85–29.29)	25.50 (23.42–28.23)	0.129
Smoking			
Non-smoker	172 (98%)	173 (99%)	0.684
Smoker	3 (2%)	2 (1%)	
Diabetics %	19 (11%)	110 (62%)	<0.0001 *
SBP (mm Hg)	136 (126–147)	142 (130–160)	0.002 *
DBP (mm Hg)	85 (79–90)	81 (72–90)	0.047 *
Hypertension (>140/90) %	38 (21%)	92 (52%)	<0.0001 *
Cholesterol (mg/dL)	195 (164–221)	162 (143–198)	<0.001 *
TG (mg/dL)	147 (109–201)	135 (107–196)	0.239
LDL (mg/dL)	116 (89–139)	95 (66–125)	<0.001 *
HDL (mg/dL)	47 (41–50)	43 (38–47)	<0.001 *
Total lipids (mg/dL)	539 (462–633)	481 (394–577)	<0.001 *
Elevated TG % (>200 mg/dL))	44 (25%)	38 (21%)	0.491
Lower HDL % (<40 mg/dL)	28 (16%)	53 (30%)	0.003 *
High Total Cholesterol % (>240 mg/dL)	22 (13%)	17 (10%)	0.473
High LDL Cholesterol % (>160 mg/dL)	23 (13%)	14 (8%)	0.154

BMI: body mass index; DBP: diastolic blood pressure; HDL: high-density lipoprotein; LDL: low-density lipoprotein; SBP: systolic blood pressure; TG: triglycerides. Data are reported as mean ± standard deviation or median (interquartile range) for continuous parameters and as number (frequency) for categorical parameters. * Statistical significance.

**Table 3:** Genotype and allele frequencies, and Hardy–Weinberg equilibrium (HWE) *p* values in both controls and cases.

Gene (Variant)	Group (*n*)	Genotype Frequency				Risk Allele Frequency (±SE)	HWE *p* Value
		Wild		Null			
*GSTM1* (rs366631)	Control (175)	0.720		0.280		-	-
	Case (177)	0.480		0.520		-	-
*GSTT1* (rs17856199)	Control (175)	0.829		0.171		-	-
	Case (177)	0.695		0.305		-	-
		I/I	I/D		D/D	D	
*ACE* (rs4646994)	Control (175)	0.263	0.514		0.223	0.480 ± 0.028	0.689
	Case (177)	0.119	0.463		0.418	0.650 ± 0.019	0.813
		T/T	C/T		C/C	C	
*AGT* (rs699)	Control (173)	0.798	0.179		0.023	0.113 ± 0.048	0.171
	Case (174)	0.460	0.448		0.092	0.316 ± 0.037	0.627
		C/C	C/T		T/T	T	
*AGT* (rs4762)	Control (175)	0.771	0.211		0.017	0.123 ± 0.047	0.801
	Case (177)	0.452	0.452		0.096	0.322 ± 0.036	0.641
		A/A	A/C		C/C	C	
*AGTR1* (rs5186)	Control (175)	0.794	0.177		0.029	0.117 ± 0.047	0.058
	Case (177)	0.441	0.446		0.113	0.336 ± 0.035	1.000
		C/C	C/T		T/T	T	
*APOE* (rs7412)	Control (175)	0.931	0.069		0.000	0.034 ± 0.052	0.639
	Case (177)	0.921	0.079		0.000	0.040 ± 0.051	0.584
		T/T	C/T		C/C	C	
*APOE* (rs429358)	Control (174)	0.736	0.236		0.029	0.147 ± 0.046	0.444
	Case (177)	0.667	0.322		0.011	0.172 ± 0.044	0.086
		G/G	C/G		C/C	C	
*APOA5* (rs3135506)	Control (169)	0.651	0.314		0.036	0.192 ± 0.044	0.901
	Case (175)	0.543	0.371		0.086	0.271 ± 0.039	0.421
		C/C	C/G		G/G	G	
*APOC3* (rs5128)	Control (173)	0.717	0.260		0.023	0.153 ± 0.046	0.972
	Case (175)	0.703	0.257		0.040	0.169 ± 0.044	0.274

**Table 4:** Odds ratios (ORs) of CAD under different association models.

Locus/Association Model	Crude OR [95% CI]	*p* Value	Adjusted OR [95% CI] ^a^	*p* Value
***GSTM1* (rs366631)**				
Recessive (null vs. wild)	2.78 [1.79–4.33]	<0.0001 **	-	-
***GSTT1* (rs17856199)**				
Recessive (null vs. wild)	2.12 [1.28–3.52]	0.0050 **	-	-
***ACE* (rs4646994)**				
Multiplicative/Allelic	2.05 [1.49–2.81]	<0.0001 **	2.14 [1.50–3.05]	<0.0001 **
Genotypic (I/D vs. I/I)	2.00 [1.10–3.62]	<0.0001 **	2.13 [1.10–4.13]	0.0004 **
Genotypic (D/D vs. I/I)	4.16 [2.18–7.93]	<0.0001 **	4.57 [2.22–9.42]	0.0004 **
Dominant (I/D + D/D vs. I/I)	2.65 [1.50–4.67]	<0.0001 **	2.83 [1.51–5.31]	0.0008 **
Recessive (D/D vs. I/D + I/I)	2.51 [1.57–3.99]	<0.0001 **	2.61 [1.55–4.39]	0.0002 **
***AGT M235T* (rs699)**				
Multiplicative/Allelic	3.61 [2.37–5.49]	<0.0001 **	3.36 [2.11–5.35]	<0.0001 **
Genotypic (C/T vs. T/T)	4.34 [2.64–7.15]	<0.0001 **	4.27 [2.46–7.40]	<0.0001 **
Genotypic (C/C vs. T/T)	6.90 [2.23–21.35]	<0.0001 **	5.03 [2.57–17.33]	<0.0001 **
Dominant (C/T + C/C vs. T/T)	4.63 [2.88–7.46]	<0.0001 **	4.36 [2.57–7.39]	<0.0001 **
Recessive (C/C vs. C/T + T/T)	4.28 [1.40–13.07]	0.0045 **	2.97 [0.88–10.03]	0.0640
***AGT T174M* (rs4762)**				
Multiplicative/Allelic	3.46 [2.29–5.21]	<0.0001 **	2.99 [1.91–4.68]	<0.0001 **
Genotypic (C/T vs. C/C)	3.65 [2.26–5.88]	<0.0001 **	3.16 [1.87–5.35]	<0.0001 **
Genotypic (T/T vs. C/C)	9.56 [2.72–33.65]	<0.0001 **	7.22 [1.87–27.83]	<0.0001 **
Dominant (C/T + T/T vs. C/C)	4.09 [2.58–6.49]	<0.0001 **	3.74 [2.08–5.76]	<0.0001 **
Recessive (T/T vs. C/T + C/C)	6.09 [1.75–21.18]	<0.0001 **	4.78 [1.24–18.36]	0.0110 *
***AGTR1 A1166C* (rs5186)**				
Multiplicative/Allelic	3.62 [2.41–5.44]	<0.0001 **	3.98 [2.54–6.26]	<0.0001 **
Genotypic (A/C vs. A/A)	4.54 [2.76–7.48]	<0.0001 **	4.72 [2.71–8.22]	<0.0001 **
Genotypic (C/C vs. A/A)	7.13 [2.57–19.74]	<0.0001 **	10.15 [3.14–32.84]	<0.0001 **
Dominant (A/C + C/C vs. A/A)	4.90 [3.06–7.85]	<0.0001 **	5.28 [3.10–8.98]	<0.0001 **
Recessive (C/C vs. A/C + A/A)	4.33 [1.59–11.82]	<0.0001 **	5.65 [1.82–17.59]	0.0011 **
***APOE* (rs7412)**				
Multiplicative/Allelic	1.16 [0.53–2.54]	0.71	1.16 [0.53–2.54]	0.87
Genotypic (C/T vs. C/C)	1.17 [0.52–2.60]	0.71	1.17 [0.52–2.60]	0.87
***APOE* (rs429358)**				
Multiplicative/Allelic	1.21 [0.81–1.82]	0.35	1.20 [0.75–1.93]	0.45
Genotypic (C/T vs. T/T)	1.51 [0.94–2.42]	0.11	1.39 [0.81–2.37]	0.36
Genotypic (C/C vs. T/T)	0.43 [0.08–2.28]	0.11	0.55 [0.08–3.84]	0.36
Dominant (C/T + C/C vs. T/T)	1.39 [0.88–2.20]	0.16	1.32 [0.78–2.23]	0.30
Recessive (C/C vs. C/T + T/T)	0.39 [0.07–2.02]	0.24	0.49 [0.10–3.31]	0.45
***APOA5* (rs3135506)**				
Multiplicative/Allelic	1.54 [1.08–2.21]	0.02 *	1.59 [1.06–2.37]	0.02 *
Genotypic (C/G vs. G/G)	1.42 [0.90–2.24]	0.02 *	1.45 [0.87–2.28]	0.06
Genotypic (C/C vs. G/G)	2.89 [1.08–7.76]	0.05 *	3.16 [1.00–9.94]	0.05 *
Dominant (C/G + C/C vs. G/G)	1.57 [1.02–2.42]	0.04 *	1.61 [0.99–2.60]	0.05
Recessive (C/C vs. C/G + G/G)	2.55 [0.96–6.73]	0.05	2.76 [0.89–8.55]	0.06
***APOC3* (rs5128)**				
Multiplicative/Allelic	1.12 [0.75–1.66]	0.58	1.04 [0.66–1.63]	0.87
Genotypic (C/G vs. C/C)	1.01 [0.62–1.63]	0.66	1.06 [0.62–1.81]	0.98
Genotypic (G/G vs. C/C)	1.76 [0.50–6.18]	0.66	0.99 [0.24–4.09]	0.98
Dominant (C/G + G/G vs. C/C)	1.07 [0.67–1.70]	0.78	1.05 [0.63–1.77]	0.85
Recessive (G/G vs. C/G vs. C/C)	1.76 [0.51–1.59]	0.37	0.98 [0.24–3.98]	0.97

^a^ Adjusted for age, blood pressure (DBP and SBP), BMI, cholesterol, diabetes, HDL, LDL, sex, and TG. * Significant at *p* < 0.05, ** Significant after Bonferroni-corrected *p*-value (0.005).

**Table 5:** Association analysis of haplotypes with CAD.

Haplotypes	Frequencies		OR [95% CI]	*p* Value	Adjusted OR [95% CI] ^a^	*p* Value
	Controls	Cases				
*AGT* rs699-rs4762						
TC	0.856	0.661	1.00 (ref)		1.00 (ref)	
CT	0.091	0.304	4.44 [2.81–7.02]	<0.0001 **	3.93 [2.39–6.48]	<0.0001 **
TT	0.032	0.018	0.89 [0.35–2.27]	0.82	0.80 [0.31–2.09]	0.65
CC	0.021	0.017	1.03 [0.35–3.03]	0.96	0.97 [0.32–3.00]	0.96
Global haplotype association				<0.0001 *		<0.0001 *
*APOE* rs7412-rs429358						
CT	0.822	0.792	1.00 (ref)		1.00 (ref)	
CC	0.143	0.169	1.23 [0.80–1.88]	0.35	1.21 [0.76–1.92]	0.43
TT	0.031	0.036	1.19 [0.47–3.03]	0.72	1.04 [0.39–2.74]	0.94
TC	0.004	0.003	1.37 [0.01–139.56]	0.89	2.17 [0.01–356.30]	0.77
Global haplotype association				0.78		0.85
*APOA5/C3* rs3135506-rs5128						
GC	0.691	0.605	1.00 (ref)		1.00 (ref)	
CC	0.156	0.227	1.62 [1.07–2.45]	0.024 *	1.86 [1.14–3.03]	0.013 *
GG	0.117	0.124	1.19 [0.71–1.98]	0.51	1.28 [0.72–2.28]	0.40
CG	0.037	0.045	1.43 [0.57–3.59]	0.44	0.88 [0.26–3.01]	0.83
Global haplotype association				0.10		0.20

^a^ Adjusted for age, blood pressure (DBP and SBP), BMI, cholesterol, diabetes, HDL, LDL, sex, and TG. * Significant at *p* < 0.05, ** Significant after Bonferroni-corrected *p*-value (0.005).

## Data Availability

Data are contained within the article.
